# Combined application of arbuscular mycorrhizal fungi and selenium fertilizer increased wheat biomass under cadmium stress and shapes rhizosphere soil microbial communities

**DOI:** 10.1186/s12870-024-05032-5

**Published:** 2024-05-03

**Authors:** Haiyang Liu, Haoquan Wang, Zhaojun Nie, Zhikang Tao, Hongyu Peng, Huazhong Shi, Peng Zhao, Hongen Liu

**Affiliations:** 1 College of Resources and Environment, Henan Agricultural University, Zhengzhou, 450046 China; 2 Key Laboratory of Soil Pollution Control and Remediation in Henan Province, Zhengzhou, 450046 China; 3 Department of Chemistry and Biochemistry, Texas Tech University, Lubbock, TX 79409 USA

**Keywords:** Cadmium; selenium, Arbuscular mycorrhizal fungi, Bacterial community, Fungal community

## Abstract

**Background:**

Selenium (Se) fertilizer and arbuscular mycorrhizal fungi (AMF) are known to modulate cadmium (Cd) toxicity in plants. However, the effects of their co-application on wheat growth and soil microbial communities in Cd-contaminated soil are unclear.

**Results:**

A pot experiment inoculation with two types of AMF and the application of Se fertilizer under Cd stress in wheat showed that inoculation AMF alone or combined with Se fertilizer significantly increased wheat biomass. Se and AMF alone or in combination significantly reduced available Cd concentration in wheat and soil, especially in the Se combined with Ri treatment. High throughput sequencing of soil samples indicated that Se and AMF application had stronger influence on bacterial community compared to fungal community and the bacterial network seemed to have more complex interconnections than the fungal network, and finally shaped the formation of specific microflora to affect Cd availability.

**Conclusion:**

These results indicate that the application of Se and AMF, particularly in combination, could successfully decrease soil Cd availability and relieve the harm of Cd in wheat by modifying rhizosphere soil microbial communities.

**Supplementary Information:**

The online version contains supplementary material available at 10.1186/s12870-024-05032-5.

## Background

As the most hazardous heavy metal, cadmium (Cd) significantly impacts human health and ecological environment [1]. Cd is absorbed from soil to plants with a high degree of mobility and eventually harm human body health through the food chain [2]. Cd can decrease biomass, result in plant yellowing, prevent photosynthesis [3, 4]. In situ stabilization or immobilized soil remediation (such as the addition of microbial agents, nutrients, and soil amendments) is considered to be the most efficient and practical measures to rehabilitate farmlands contaminated by heavy metals [5].

Selenium (Se) is a crucial microelement for living body, which can boost immunity, fend off disease, and delay the aging process [6]. Although Se is not essential for plants, some researchers have found that Se can promote plant growth and enhance resistance to oxidative stress. The Se also plays an important role in enhancing photosynthesis and facilitating the repair of cell damages [4, 7]. Furthermore, some researches have indicated that Se may reduce Cd damage to plants. For example, in both hydroponic and soil experiments, Se has shown potential in mitigating the negative effects of Cd on plants, especially by reducing the translocation and accumulation of Cd in grains [8, 9]. Ahmad et al. [10] found that application of Se fertilizer in rice seedlings could reduce Cd transfer to shoot. Similarly, several studies revealed Se can change the chemical form of Cd and decrease its availability in soil [11, 12].

Arbuscular mycorrhizal fungi (AMF) are a group of important symbiotic microorganisms, which can promote plant growth by promoting nutrient uptake and carbohydrate utilization [13, 14]. The mechanism of AMF reducing plant Cd uptake mainly includes: (1) altering the expression level of heavy metal transporter gene [15, 16]; (2) enhancing the absorption of nitrogen and phosphorus by plants, consequently increasing plant biomass and reducing heavy metal concentrations through dilution effect [17]; (3) binding heavy metals to fungal mycelium, decreasing plant accessibility to heavy metals [18]; (4) lowering Cd concentration in cell walls and vacuoles, influencing Cd absorption and altering its chemical form [19].

It has been reported that AMF and plant rhizosphere microorganisms can enhance plant tolerance to abiotic stresses and promote plant growth in contaminated soil [20]. The application of Se fertilizer to soil has been shown to improve the tolerance of plants to heavy metal stress by controlling rhizosphere processes and the formation of rhizosphere microbial communities [21]. AMF may stimulate microbial metabolic activity through nutrients released in the mycelial circle similar to those secreted by plant roots [22], which in turn can affect the adsorption and desorption process of heavy metals in soil [23]. Based on recent findings, it has been revealed that the combination of AMF and Se fertilizer has the potential to influence the composition of bacterial and fungal communities and functional properties [24–26]. However, the effects of the application of AMF and Se on Cd availability and soil microorganisms in wheat at seedling stage under Cd stress are still unclear. Thus, this study was to investigate the effects of AMF combined with Se fertilizer on wheat growth, Cd bioavailability and rhizosphere microbial communities under Cd stress. We hypothesized that the combination of AMF and Se fertilizer would enhance wheat growth and reduce Cd accumulation by shaping soil microbial community.

## Results

### Effects of Se and AMF on wheat growth

Compared to the CK, the wheat plants treated with selenium or AMF had better growth (Fig. S1). For details, as shown in Table 1, compared to the CK, the AMF and Se + AMF treatments remarkably increased wheat biomass, while Se treatment decreased wheat biomass, the shoot dry weight was enhanced by 72.54% and 67.61% with the inoculation of Fm and Ri, respectively. (*P* < 0.05) The root dry weight also showed a significant increase of 92.68% and 121.95% in the respective treatments. In addition, compared to the Se treatments, the combination of Se and AMF significantly increased wheat biomass (*P* < 0.05). Notably, the Se + Ri treatment showed a remarkable increase in both shoot and root dry weight, with 61.26% and 82.93% increment respectively (Table 1).

**Table 1:** Shoot and root dry biomass of wheat

Treatment	Shoot dry weight/g pot^−1^	Root dry weight/g pot^−1^
CK	1.42 ± 0.07c	0.41 ± 0.05d
Se	1.15 ± 0.1d	0.43 ± 0.06 cd
Fm	2.45 ± 0.09a	0.79 ± 0.03b
Ri	2.38 ± 0.05a	0.91 ± 0.15a
Se + Fm	1.86 ± 0.22b	0.51 ± 0.03c
Se + Ri	2.29 ± 0.26a	0.75 ± 0.04b

The data presented in the table is mean values of three replicates of each treatment along with standard deviation (SD). Different small letters indicate the significant difference among the treatments at *P *< 0.05. CK: control; Se: addition of 5 mg kg^-1^ Se fertilizer; Fm: inoculation of Fm; Ri: inoculation of Ri; Se + Fm: combined addition of Se and Fm and Se + Ri: combined addition of Se and Ri

### Effects of Se and AMF on cd concentration in wheat tissues and soils

**Fig. 1:**
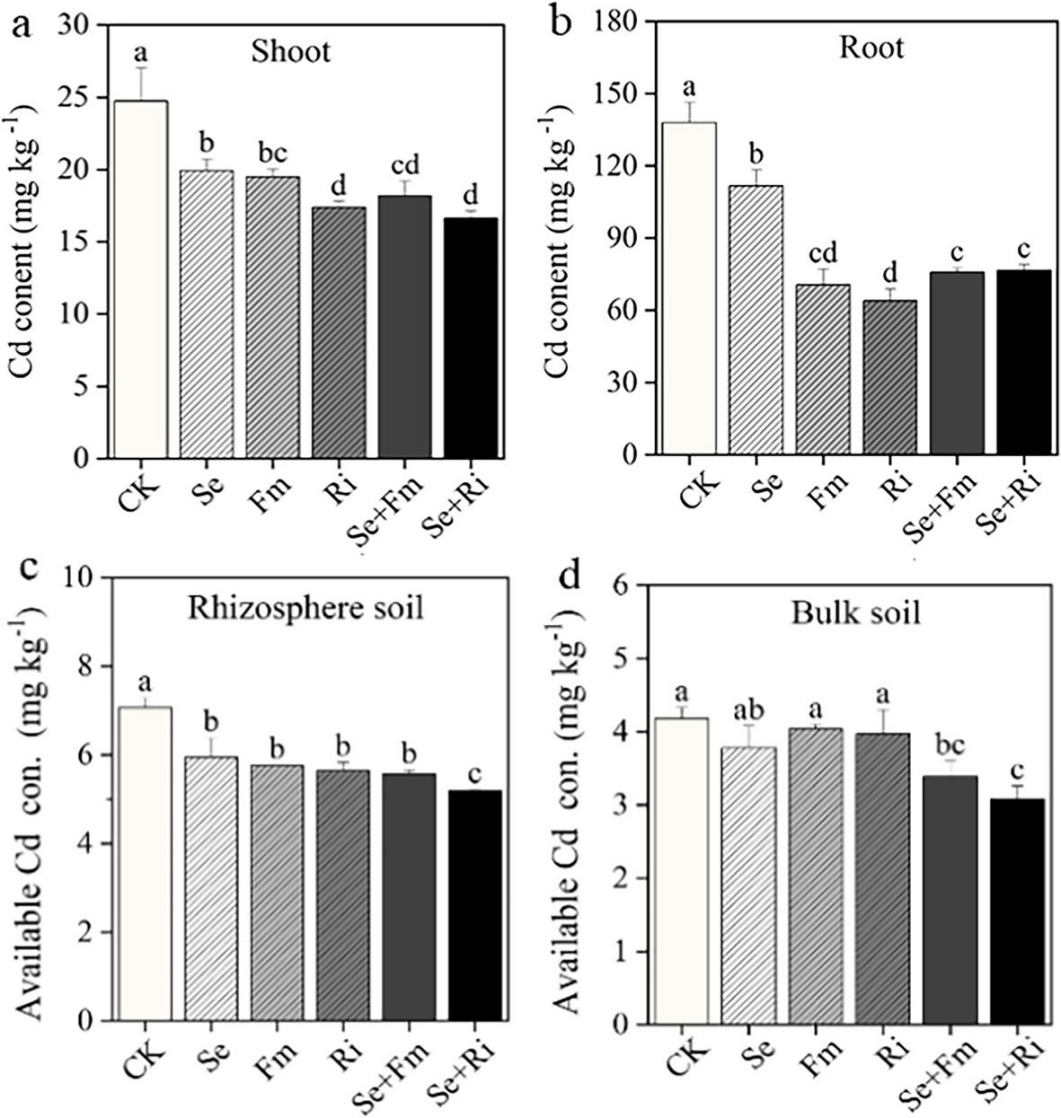
Effects of different treatments on total Cd concentration in shoot (**a**) and root (**b**), and available Cd concentration in rhizosphere (**c**) and bulk soils (**d**) of wheat. The data shown in the figure are the mean and standard deviation (SD) of three replicates for each treatment. Significant changes between treatments at *P* < 0.05 were denoted by various lowercase letters above the bars. CK: control; Se: addition of 5 mg kg^-1^ Se fertilizer; Fm: inoculation of Fm; Ri: inoculation of Ri; Se + Fm: combined addition of Se and Fm and Se + Ri: combined addition of Se and Ri

The application of Se and AMF alone or in combination could effectively reduce total Cd concentration in wheat tissues (Fig. 1a and b). In the CK treatment, the concentrations of total Cd in both shoot and root were the highest. Conversely, the Se + Ri treatment displayed the lowest concentration of total Cd in the shoot (Fig. 1a), while the lowest Cd concentration in the root was observed in the Ri treatment (Fig. 1b). Compared to the CK, the shoot’s total Cd concentration in Fm and Ri treatments was decreased by 21.23% and 29.72%, respectively, and the root’s total Cd concentration was reduced by 48.87% and 53.71%, respectively. Both Se and two AMF treatments significantly decreased total Cd concentration in wheat. For instance, total Cd concentration in the shoot decreased from 24.73 mg kg^-1^ in the CK treatment to 18.18 mg kg^-1^ in the Se + Fm treatment and 16.64 mg kg^-1^ in Se + Ri treatment (Fig. 1a). However, compared to the Se treatment, the Ri treatment showed a greater reduction in root total Cd concentration significantly than the Fm treatment (*P* < 0.05).

The CK treatment had the highest available Cd concentrations in both rhizosphere and bulk soils, while the Se + Ri treatment showed the lowest available Cd concentrations (Fig. 1cd). In the rhizosphere soil, the application of Se and AMF alone could significantly decrease available Cd concentration (*P* < 0.05). In comparison to the CK treatment, the application of Se reduced available Cd concentration by 14.61%, and inoculating Fm and Ri reduced available Cd concentrations by 17.50% and 19.94%, respectively. The combination of Se + Fm or Se + Ri decreased available Cd concentrations by 21.52% and 26.83%, respectively (Fig. 1c). In the bulk soil, compared to the CK treatment, the presence of Fm and Se alone did not have a significant impact on the available concentration of Cd (*P* > 0.05), And the combination of Se and AMF resulted in a remarkable reduction of available Cd concentration. Particularly, Se + Ri treatment exhibited the most substantial decrease in available Cd concentration (Fig. 1d). In addition, the total Cd concentration in the rhizosphere soil was significantly decreased by all treatments when compared to the CK (*P* < 0.05), and Se + Ri treatment was particularly effective, showing a reduction rate of 25.61% (Fig. S2a). In the bulk soil, Se and Fm treatments reduced the total Cd concentration compared to CK, but it’s not significant. The combined with Se and AMF or inoculated with Ri alone significantly decreased the total concentration of available Cd, especially in the Se + Ri treatment which reduced 24.29% (Fig. S2b).

### Effects of Se and AMF on chemical form of cd in rhizosphere soil

**Fig. 2:**
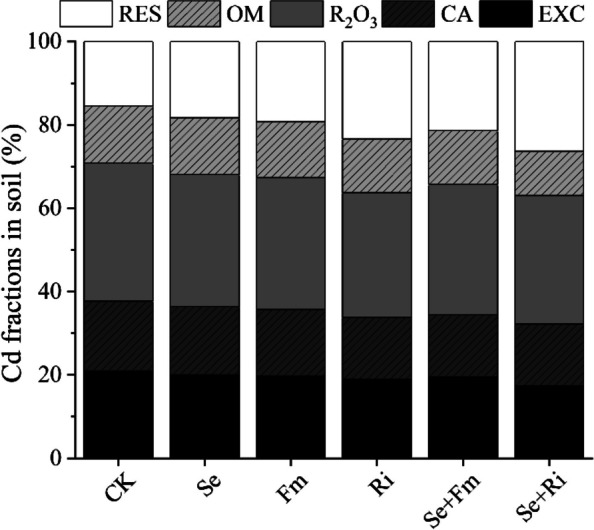
Effects of different treatments on the chemical forms of Cd in rhizosphere soil. EXC: exchangeable form; CA: carbonate - bound; R_2_O_3_: iron (Fe) - manganese (Mn) oxide - bound; OM: organic - bound and RES: residual form. All other designations are the same as those in Fig. 1

The application of Se or AMF exhibited a pronounced impact on the composition of Cd chemical forms in soil (Fig. 2). Application of Se fertilizer and AMF led to a significant decrease in EXC - Cd, CA - Cd and R_2_O_3_ – Cd, along with a significant increase in OM - Cd and RES - Cd in soil compared to the CK treatment (*P* < 0.05). The Se + Ri treatment demonstrated a greater reduction in Cd availability compared to the Ri treatment. For instance, R_2_O_3_ - Cd decreased from 12.33% in the Ri treatment to 9.82% in the Se + Ri treatment, and RES – Cd increased from 28.13% in the Ri treatment to 74.06% in the Se + Ri treatment (Fig. 2). The Se + Ri treatment had the most significant effect in decreasing soil available Cd concentration compared to the CK (*P* < 0.05). For example, EXS - Cd, CA - Cd and R_2_O_3_ – Cd decreased by 92.56%, 68.47% and 66.29%, respectively, while OM - Cd and RES - Cd increased by 123.81% and 29.22%, respectively. Compared to the treatment with Se alone, the Se + AMF significantly decreased the concentrations of EXS - Cd, CA - Cd, and OM - Cd (*P* < 0.05). The Se + Ri treatment showed a more significant effect, resulting in reduction rates in the above Cd forms of 61.76%, 38.67%, and 23.97%, respectively (Fig. 2).

### Effects of Se and AMF on Se concentration in wheat tissues and soils

**Fig. 3:**
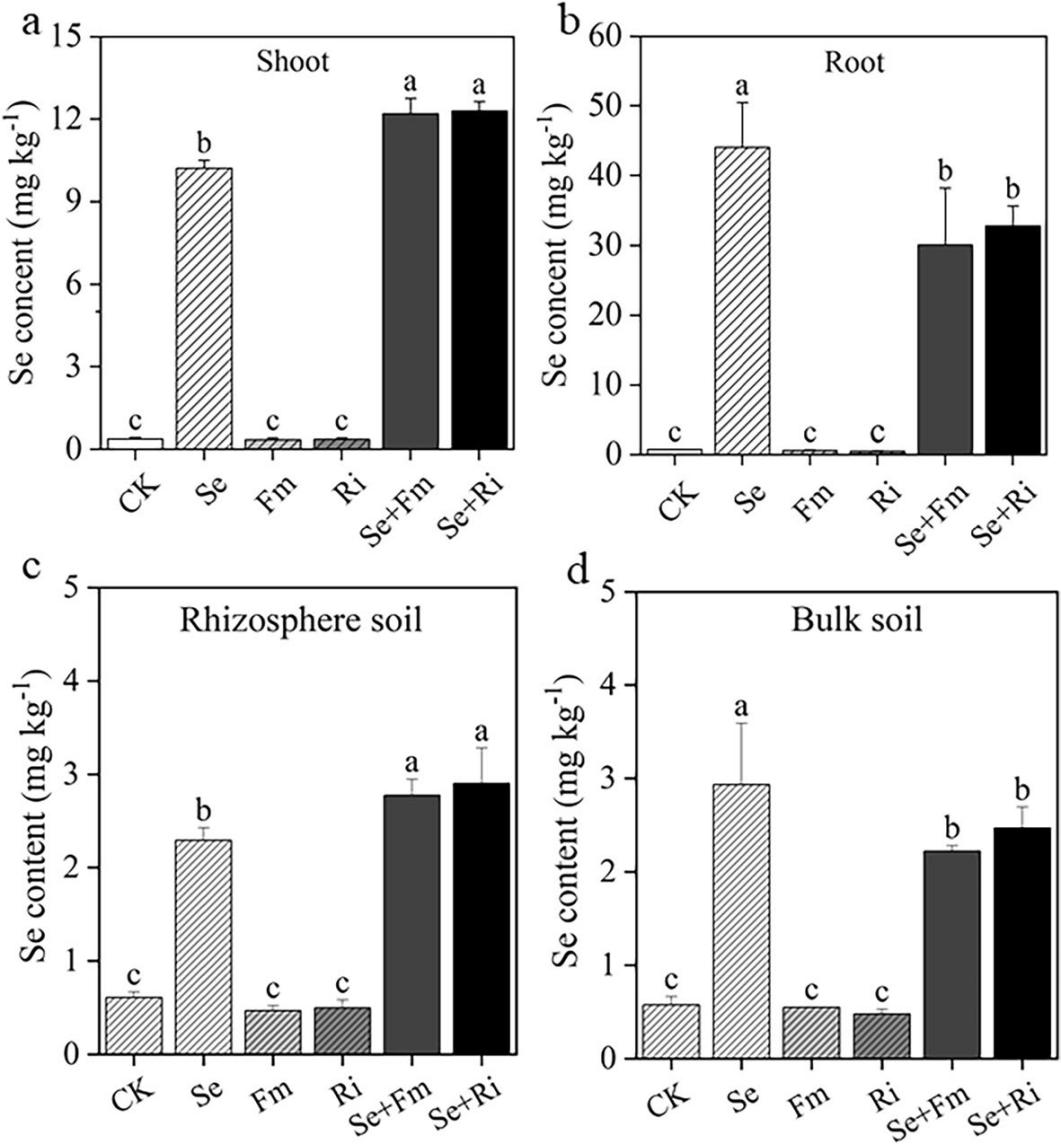
Effects of different treatments on total Se concentrations in shoots (**a**), roots (**b**), rhizosphere (**c**) and bulk soils (**d**) of wheat. All other designations are the same as those in Fig. 1

Compared to the Se treatment, the combination with Se and AMF remarkably reduced total Se concentration in root and increased total Se concentration in shoot of wheat (Fig. 3a and b). For instance, the total Se concentration in the shoot of Se + Ri treatment reached the highest, increased by 20.23% compared with Se treatment. However, Se + Fm and Se + Ri treatments decreased total Se concentration in root by 31.61% and 25.68%, respectively (Fig. 3b). The total Se concentration in the rhizosphere soil was increased by the combined application of Se and AMF, with the Se + Ri treatment showing a significant increase of 26.87% (Fig. 3c). In the bulk soil, the total Se concentrations showed significant decreases from 2.92 mg kg^-1^ in the Se treatment to 2.22 mg kg^-1^ in the Se + Fm treatment and 2.47 mg kg^-1^ in the Se + Ri treatment (Fig. 3d).

### Effects of Se and AMF on soil bacterial and fungi communities

Regarding the bacterial community, the inoculation of AMF alone increased Chao 1, Shannon and Simpson indexes compared to the CK treatment (Fig. S3a-c). The application of Se alone increased Chao 1 index (Fig. S3a). However, compared with the CK treatment, when Se and AMF were combined, the Chao 1 index showed a decrease (Fig. S3a). The combination of Se and AMF decreased the Shannon and Simpson indexes compared to the inoculation of AMF alone (Fig. S3b and c). As for the fungal community, the Se or Fm treatments significantly improved the Chao1 index (Fig. S3d). The Se + Ri treatment remarkably increased the Shannon index compared to the CK treatment (*P* < 0.05) (Fig. S3e). However, no discernible variation in the Simpson index was appeared among all treatments (*P* > 0.05) (Fig. S3f).

**Fig. 4:**
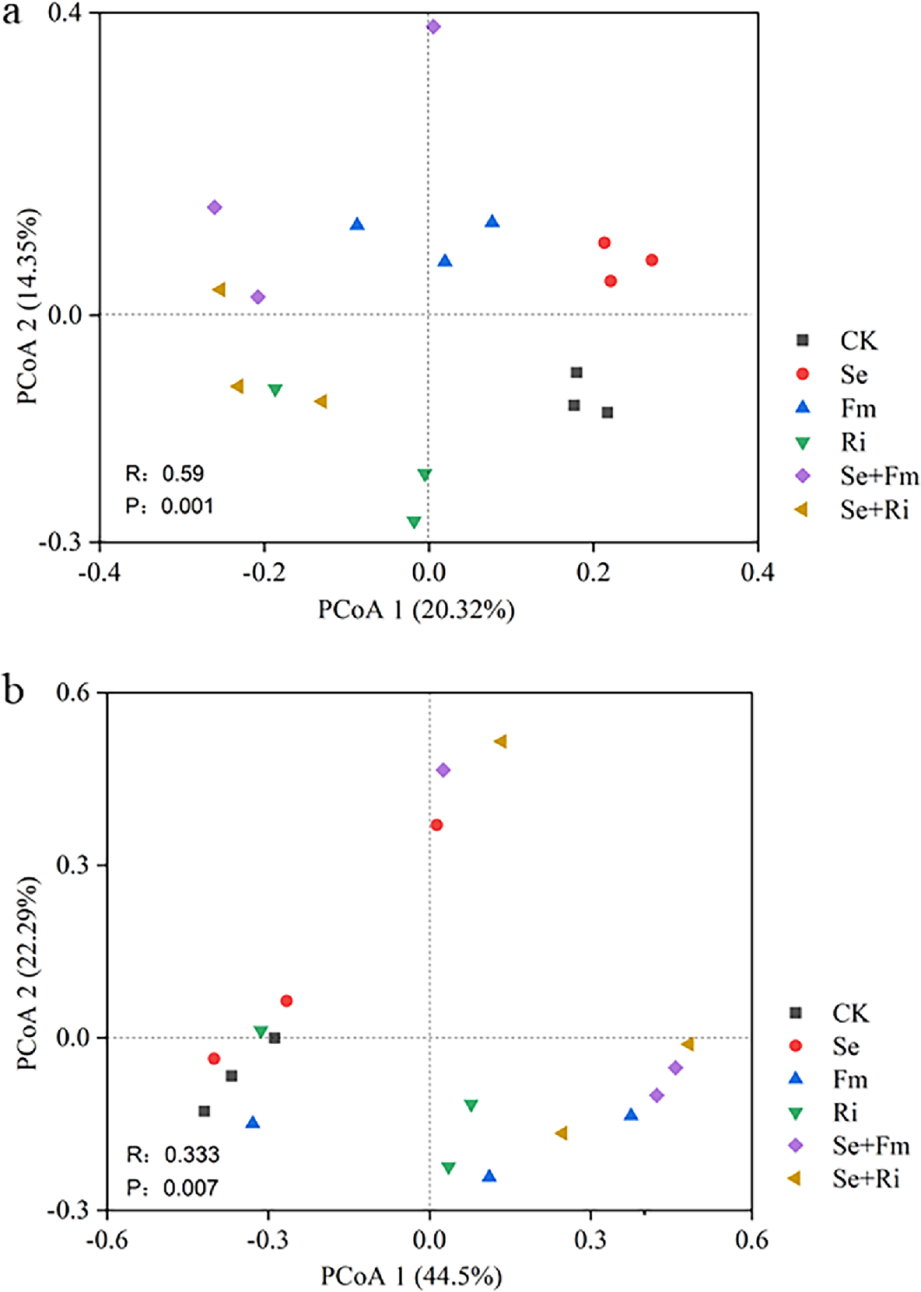
Principal coordinate analysis (PCoA) of bacterial (**a**) and fungal (**b**) communities based on the Bray-Curtis distance. The results of similarity analysis (ANOSIM) are expressed as P and R values

PCoA plots were assessed by the variations in microbial communities among treatments (Fig. 4). In the bacterial community, there was an obvious separation from the CK to the other treatment. The first axis showed a clear separation between CK and Ri, CK and Fm treatments, and a significant difference between Se and Se + AMF treatments. On the second axis, there was a clear separation between CK and Se treatments, Fm and Ri treatments. The bacterial communities were also different between Ri and Se + Fm treatments, Ri and Fm treatments, but no difference between Ri and Se + Ri treatments (Fig. 4a). For the fungal community, the first axis showed a significant separation between CK and Se + AMF treatments, but no differences were showed in the other treatments (Fig. 4b) In addition, the ANOSIM analysis revealed the very significant difference in the bacterial community (*R* = 0.59, *P* = 0.001), but significant difference in the fungal community (*R* = 0.33, *P* = 0.007) (Fig. 4).

**Fig. 5:**
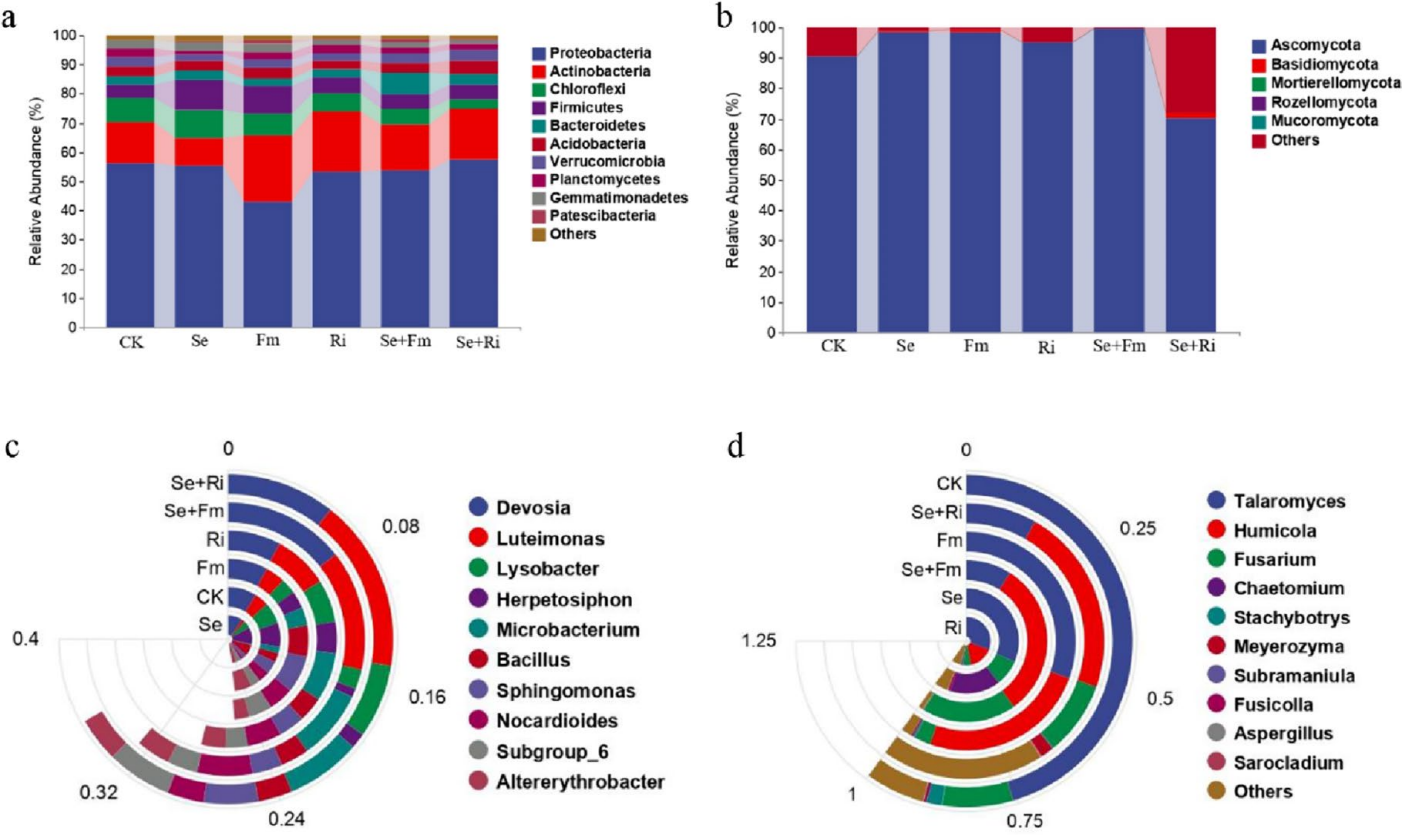
Effects of different treatments on the community compositions of bacteria (**a** and **c**) and fungi (**b** and **d**) at the phylum (**a** and **b**) and genus levels (**c** and **d**)

In all soil samples, the dominant bacterial phyla were *Proteobacteria*, *Actinobacteria*, *Chloroflexi*, *Fimicutes*, *Bacteroidetes* and *Acidobacteria* accounting for more than 87% of the total bacterial sequences (Fig. 5a). The relative abundance of *Proteobacteria* was higher only in the Se + Ri treatment, which increased from 56.21% in the CK treatment to 57.27% in the Se + Ri treatment. Se and AMF treatments increased the relative abundance of *Firmicutes*, particularly in the Se treatment where it increased from 4.56% in the CK treatment to 10.50% (Fig. 5a). Moreover, both AMF and Se + AMF treatments enhanced the relative abundance of *Actinobacteria*, and Fm treatment increased the relative abundance of *Actinobacteria* to 22.57% from 14.09% in the CK treatment (Fig. 5a). With regard to the fungal community, Ascomycota and Basidiomycota were the dominant phyla in all samples, comprising over 95% of the fungal sequences (Fig. 5b). In comparison with the CK, the relative abundance of Ascomycota in the Se, Fm, Ri and Se + Fm treatments was higher, increasing by 8.74%, 8.68%, 4.86% and 9.95%, respectively. However, the relative abundance of Ascomycota in the Se + Ri treatment was decreased by 22.37% (Fig. 5b). Meanwhile, the relative abundance of Basidiomycota reached a maximum of 1.63% in the Se + Ri treatment, while was absent in the CK and Se + Fm treatments (Fig. 5b).

*Devsia* and *Luteimonas* were the dominant bacteria genera in soil samples, accounting for 23.31 − 54.95% (Fig. 5c). All treatments except AMF inoculation alone enhanced the relative abundance of *Devsia*. In the Se and Se + Fm treatments, the relative abundance of *Devosia* increased to 19.51% and 27.36%, respectively (Fig. 5c). The Se + Fm treatment had the highest relative abundance of *Microbacterium*, and the relative abundance of *Herpetosipho* reached a remarkable 21.9% in the Se treatment. In comparison to the CK, the Se + Ri treatment enhanced the relative abundance of *Lysobacter* and *Luteimonas*. However, the relative abundances of *Herpetosiphon* and *Altererythrobacter* were reduced in the Se + Ri treatment (Fig. 5c). Among the fungal community, *Talaromyces* was the dominant genus, accounting for 13.92 − 75.89% (Fig. 5d). Compared to the CK treatment, the relative abundance of *Talaromyces* was reduced in all treatments. The Se + Fm and Se + Ri treatments showed the most significant effects, resulting in the relative abundance of *Talaromyces* decreases to 15.18% and 13.92% respectively. The relative abundance of *Humicola* was the highest in the Se + Fm treatment, increasing from 41.43% in the Fm treatment to 51.61% in the Se + Fm treatment (Fig. 5d). In addition, compared to the CK treatment, the AMF treatments reduced the relative abundance of *Fusarium* but showed significant increases in the Se and Se + AMF treatments. The Se + Fm treatment had the highest relative abundance of *Fusarium*, increasing from 4.00% in the Fm treatment to 31.31% in the Se + Fm treatment (Fig. 5d).

### Taxonomic biomarkers of soil microbial communities in different treatments

**Fig. 6:**
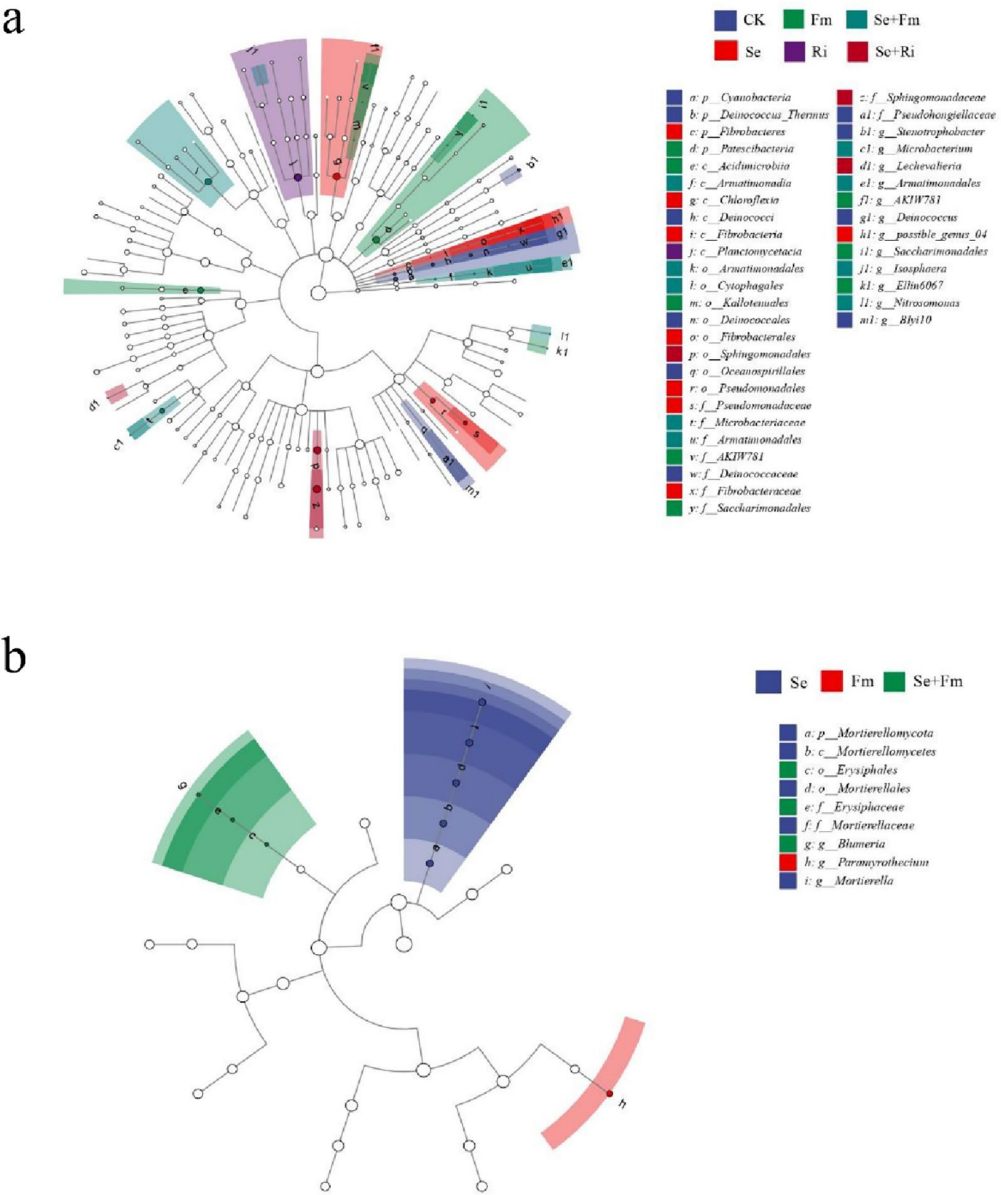
LEfSe cladograms showing taxa with different abundance values. Taxonomic cladogram obtained from LEfSe analysis of bacterial and fungal communities. Only taxa satisfying an LDA significance threshold 2.0 for bacterial community (**a**) and fungal community (**b**) are displayed. Seven rings represent domain (innermost), phylum, class, order, family, genus, and species (outermost), respectively

 The bacterial and fungal groups with an LDA score of 39 and 9 greater than 2.0 were used as biomarkers between treatments (Fig. 6a and S4a). For the bacterial community, the *Deinococcus, Srenotrophobacter, Oceanosp, irillales, Blyi10* and *Deinococcales* were more enriched in the CK treatment. In addition, *Chloroflexi, Fibrobacter, Pseudomonas, Mortierelomycota, Mortierellomycetes, Mortierellales, Mortierelaceae* and *Mortierella* were identified as biomarkers in the Se treatment. The genus-level biomarkers of *Acidimicrobiia, AKIW781, Kallotenualesp, Palescibacteria, Saccharimonadalesg, Saccharimonadales* and *Ellin6067* were found in the Fm treatment. Only one species of *Planctomycetacia* class was dominant in the Ri treatment. *Cytophagales, Microbacteriaceae, Microbacterium, Isosphaera, Nitrosomonas, Armatimonadia* and *Armatimonadales* were significantly enriched in the Se + Fm treatment, *Sphingomonadales, Sphingomonadaceae* and *Lechevalieria* were identified as biomarkers of the Se + Ri treatment (Fig. 6a and S4a). As for the fungal community, only the Se, Fm and Se + Fm treatments showed biomarkers. In the Se treatment, *Mortierellomycetes, Mortierellaceac, Mortierellacac* and *Mortierella* at the level of *Mortierellomycota* were all marker groups. In the Fm treatment, only *Paramyrothecium* was the most significant, while *Erysiphaceae* and *Blumeria* at the level of *Erysiphales* were identified as biomarkers in the Se + Fm treatment (Fig. 6b and S4b).

### Microbial module analysis

**Fig. 7:**
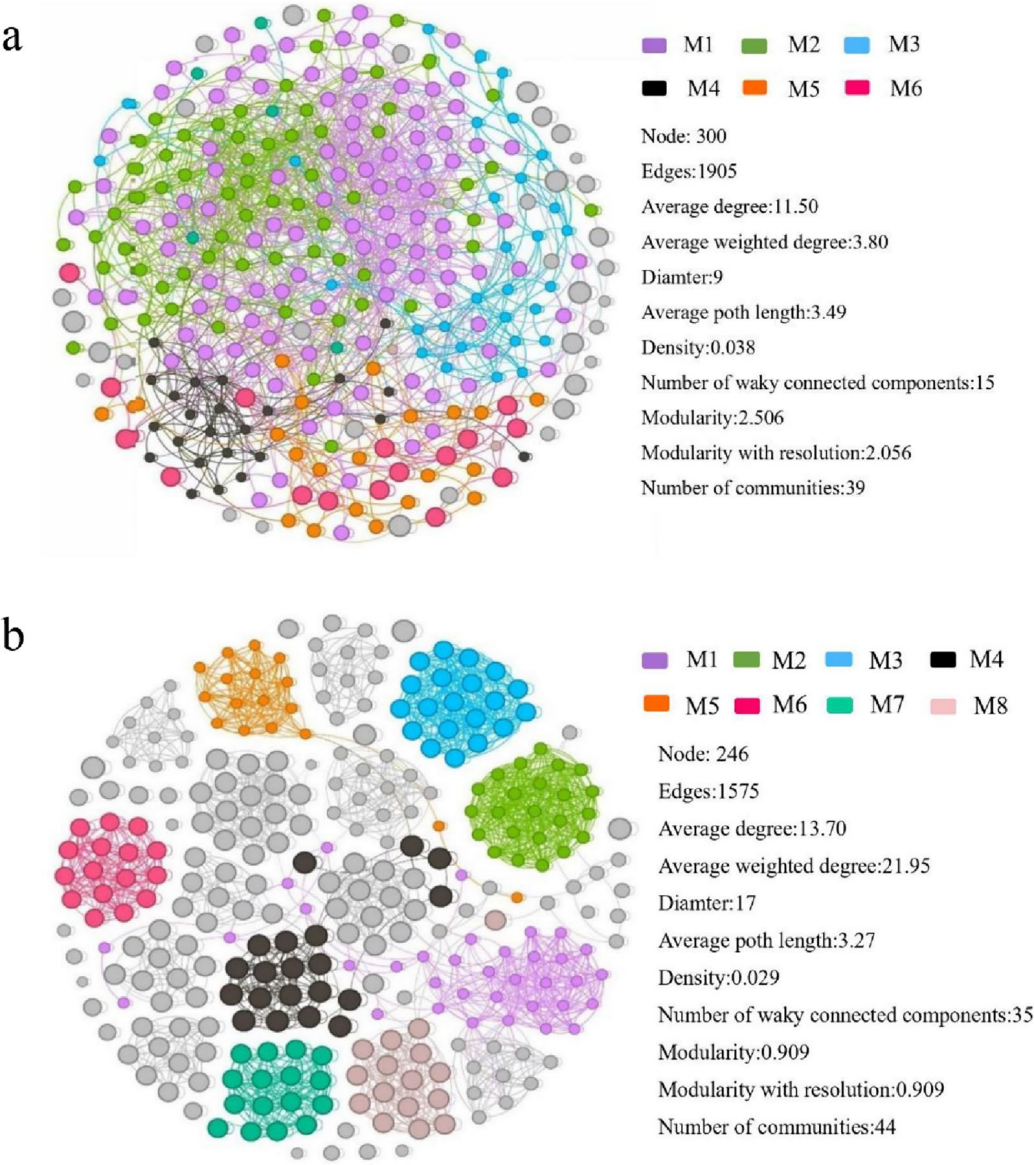
Co-occurrence networks of ASVs from different treatments for bacteria (**a**) and fungi (**b**). Nodes of the same color belong to the same module. An edge (line) represents a relationship between two substances

Through network analysis, multiple modules were detected in the bacterial and fungal communities, respectively (Fig. 7). Bacterial networks have more nodes (300) and edges (1905) than fungal networks which have 246 nodes and 1575 edges. The average length (3.49) and density (0.038) of bacterial networks were also higher than fungal networks, indicating that bacterial networks appear to be more complex and interconnected than fungal networks. In bacterial communities, there is more overlap in module composition, and M1 contributes more to bacterial modules than M2, M3 and M4. M1 and M2 mainly include *Proteobacteria* and *Actinobacteria*. M1 is dominated by *Gammaproteobacteria* and *Alphaproteobacteria*, and M2 is represented by *Rhizobiales* and *Sphingomonadales* (Fig. 7a). Within the fungal community, each module clustered independently in the network. The contribution rate of M1 reached 11.67%, primarily comprised *Sordariales* and *Hypocreales*, both belonging to the Ascomycota phylum, which accounted for 32.35% and 23.53% of the module, respectively. M2 predominantly consisted of *Fusarium* within the Ascomycota phylum (Fig. 7b).

## Discussion

### Impact of Se and AMF on wheat growth

Our results found the application of 5 mg kg^-1^ Se significantly decreased shoot dry weight (Table 1). The concentration of Se for plant growth is not as high as possible [27, 28]. For example, a previous study has shown that applying 5 mg kg^-1^ Na_2_SeO_3_ could improve wheat yield under the Cd levels of 6 mg kg^-1^, but higher levels of Na_2_SeO_3_ at 10 mg kg^-1^ or above could reduce grain yield [29]. In addition, Rani et al. [30] shown that the application of > 4–5 mg kg^-1^ Se has adverse effects on dry matter yield of soybean, corn and wheat, while the tolerance threshold of rice is as high as 10 mg kg^-1^. The threshold of Se tolerance of different crops was different, and the dry matter yield decreased significantly after exceeding the threshold. The decrease in dry matter and yield is attributed to the substitution of Se for sulfur in essential organic compounds and leads to the disruption of normal biochemical reactions and enzyme function [31]. However, our results also found AMF alone or combined with Se fertilizer could promote wheat plant growth compared to the CK (Table 1). Studies have shown that AMF can alleviate Cd stress in wheat by fixing Cd and reducing the production and removal of reactive oxygen species [32]. AMF can alleviate Se stress by promoting the growth and nutrient absorption of maize under high Se stress [33]. Therefore, we suggested that the inoculation of AMF can offset the negative impacts of higher Se content on wheat growth, ultimately reducing Cd toxicity through the increased dilution of Cd mass.

### Impact of Se and AMF on Cd and Se concentrations

It has been shown that AMF inoculation or Se application could decrease soil Cd concentration and help crops alleviating Cd stress [12, 34]. Our data supports these findings, demonstrating that both single and combined use of Se fertilizer and AMF could significantly reduce available Cd concentration in both shoots and roots of wheat. AMF affects Cd uptake indirectly or directly mediated by alterations in the bacterial community composition within rhizosphere soil and changing the abundance of Cd transporters [35]. AMF was also shown to associate symbiotically with soil microorganisms to restrict the movement and uptake of Cd by wheat [36]. Se fertilizer application can effectively hinder Cd absorption and translocation [37]. According to our findings, the combined application of Se fertilizer and AMF showed a more pronounced impact, which indicated that Se fertilizer and AMF inoculation may work together synergistically to convert soil Cd into a less absorbable form, ultimately reducing Cd uptake by wheat (Fig. 1 and S2). In addition, soil pH showed significant increases in the Se + AMF treatment (Table S1). Previous study found that the increase in soil pH induced the formation of hydroxide precipitates of Cd, which in turn reduced the efficiency of Cd [38]. Our findings further proved the mechanism through which increasing pH by Se and AMF amendment can lessen the activity of Cd in the soil. In addition, our study observed that the application of 5 mg kg^-1^ Se did not alleviate Cd stress of wheat but decrease soil available Cd content (Table 1 and Fig. 1). This was consisted with a previous study which showed that the application of 2.5 mg kg^-1^ Se could reduce the growth of wheat under Cd stress, but also reduced the availability of Cd in soil [39]. This may because that although higher Se content has an inhibitory effect on plant growth, Se and Cd themselves have antagonistic effects, which will change the soil Cd composition and further reduce the absorption of Cd by plants.

The Se + AMF treatment could increase Se transfer to rhizosphere soil and increase Se uptake by wheat shoot (Fig. 3). A previous research have indicated that inoculation with AMF can transfer beneficial elements to the host root system through symbiotic hyphae and improve the utilization of beneficial elements [40]. Inoculation with AMF can improve Se uptake by regulating the root transcriptome [41]. Furthermore, our study discovered that in the Se + AMF treatment, the shoot has the highest total Se concentration and the lowest available Cd concentration (Figs. 1 and 3). Shao et al. [42] found a negative correlation between available Se and Cd concentration, and Se can enhance the antioxidant level and act as a chelator to mitigate Cd-induced damage in plants. Application of Se can regulate lignin synthesis, modulate the expression of genes related to toxicity resistance of Cd, and reduce Cd permeability into plant cells [43]. Hence, the combined use of Se and AMF presents a more effective approach to mitigating the harmful effects of Cd on wheat.

### Response of soil microbial communities to Se and AMF application

Both single and co-application of Se fertilizer and AMF changed abundance and diversity of soil microbes, but the rhizosphere bacterial community responded more strongly than fungi community to Cd (Fig. S3). As for bacterial communities, the presence of Cd was found to have a detrimental impact on bacterial diversity. However, there was no reduction in the Shannon and Simpson indexes with the inoculation AMF. This suggests that the inoculation with AMF may have mitigated the stress caused by high concentrations of Cd (10 mg kg^-1^) on rhizosphere bacteria (Fig. S3a). Similar results have been observed in other Cd-contaminated soils, where inoculation with AMF significantly increased bacterial richness and diversity in the rhizosphere, indicated that the mycelial structure of AMF could detoxify heavy metals by chelating and segregating them, thus altering the distribution of heavy metals [44]. However, the combined application of Se and AMF decreased bacterial α-diversity. This could be because Se and AMF may shape specific microbes to resist Cd stress. For fungal communities, there was no statistical difference between Se or AMF for the Simpson index (Fig. S3f). The fungi were lower vulnerable to heavy metals [45] and fungal populations seem to be more stable in terms of α-diversity compared to bacterial communities [46]. It may be that contaminants can alter some local microbial populations by exerting selective pressure, but there will be others unchanged. Meanwhile, we discovered that bacteria had a higher Chao 1 index than fungi (Fig. S2). Liang et al. [47] are consistent with our experimental results, which showed that bacteria may play a more important role than fungi.

PCoA results indicated that AMF and Se fertilizer had a greater impact on bacterial communities compared to fungal communities (Fig. 4). This finding supports previous researches indicating that AMF inoculation affected soil microbial communities, with fungal communities demonstrating higher resilience to treatment variations than bacterial communities, as the latter exhibit greater stability in their response to pollutants compared to bacteria [42, 48]. Moreover, we also found that the simultaneous use of Se and AMF had a more significant impact on the bacterial community when compared to using Se fertilizer or AMF separately (Fig. 4). These findings are consistent with previous discoveries found by Chen et al. [26], which indicated that the combined use of AMF and Se fertilizer can effectively alter the bacterial community composition in the rhizosphere soil.

The co-application of Se fertilizer and AMF significantly increased the abundance of *Actinomycetes* (Fig. 5a), which are essential for controlling the soil environment and nutrient cycling [49]. A previous study has shown that the combination of *Actinomycetes* and *Mucor* has capacity to clean up polluted soil, specifically in the case of the zinc, lead, and manganese compounds [50]. The Ri and Se + Ri treatments increased the relative abundance of *Lysobacter* (Fig. 5c). This finding is consistent with a previous study indicating that *Lysobacter* can better promote plant development by affecting several plant chemicals like indole–3-acetic acid (IAA) and 1-Aminocyclopropane-1-carboxylic acid (ACC) [51]. This also explains that Ri fungi in this experiment can promote wheat growth (Table 1). In addition, compared to the CK treatment, the AMF and Se + AMF treatments enhanced the relative abundance of *Altererythrobacter* and *Microbacterium* (Fg. 5c). Meanwhile, *Microbacteriaceae* and *Microbacterium* were also found to be the biomarker species in the Se + Fm treatment (Fig. 6 and S4a). *Altererythrobacter* has the potential to remediate heavy metal-contaminated environments, as it encodes 10 genes that confer resistance of heavy metals [52]. *Microbacterium* have been shown to affect the mobility resist different heavy metals in contaminated soil [53]. In term of fungal community, Ascomycota and *Fusarium* had the highest relative abundance in the Se + Fm treatment (Figs. 3d and 4b), in particular, members of *Leotiomytes* at the level of Ascomycota were also found to be significantly enriched in Se + Fm treatment (Fig. 5b and S4b). Previous studies have shown that Ascomycota have complex spores and can accumulate heavy metals in the body to withstand heavy metal stress [54, 55]. *Fusarium* exhibit a capacity to bind and eliminate heavy metals [34]. These previous reports mechanistically support our findings that Se + AMF treatment enhance Cd resistance in wheat.

In addition, the network analysis further supported the distinct patterns of bacteria and fungi in the rhizosphere soil. Specifically, it revealed that the bacterial community exhibited a more complex and closely interconnected network module, while the fungal community displayed a more clustered and independent network structure (Fig. 7). Previous studies have shown that communities with close symbiosis have low stability and are more susceptible to disturbance [56]. In this study, bacterial communities were more sensitive to environmental changes, especially *Proteobacteria* and *Actinobacteria* in M1 and M2, which were closely associated and more sensitive to AMF inoculation (Figs. 5 and 7). *Proteobacteria* can survive in extreme environments (such as high concentration of heavy metals) through carbon cycling and nitrogen fixation, and promote plant growth systems [57]. *Actinobacteria* adapt to heavy metals in soil by reducing heavy metal concentrations through powerful secondary metabolism [58]. Among fungal communities, *Sordariales* and *Hypocreales* (*Sordariomycetes* at class) are common fungal communities in M1. *Sordariomycetes* (*Ascomycota* at phylum) demonstrates environmental tolerance and plays a crucial role in sustaining agricultural ecosystems by withstanding Cd stress [59]. Zhao et al. [60] found that *Hypocreales* is a cellulose hydrolase-producing bacterium that facilitates the degradation of cellulose into polysaccharides and other substances. This process promotes the decomposition of organic matter in soil, thereby enhancing plant growth and development.

## Conclusion

This study revealed the effects of AMF inoculation and Se fertilizer on alleviating Cd stress of wheat by reducing available Cd concentration, increasing Se concentration and influencing microbial communities. Compared to other treatments, the combination of Se fertilizer with *Rhizophagus intraradices* had a more marked effect to reducing available Cd concentration in wheat shoot. The application Se or AMF alone or in combination effectively reduced soil available Cd concentration, and the combination of Se and *Rhizophagus intraradices* showed better positive effect. In addition, the application of Se and AMF changed soil microflora, particularly bacterial community, resulting in bacterial networks that are more tightly connected than fungal networks and enriched some specific populations that may provide resistance to Cd toxicity. These results demonstrated that the combined application of *Rhizophagus intraradices* and Se fertilizer is an effective measure to alleviate Cd stress in wheat. However, this study used pot experiments by simulating Cd pollution, and additional studies are necessity to evaluate the impact of AMF and Se on Cd remediation at natural Cd contaminated sites.

## Materials and methods

### Soil pretreatment

Soil samples used for this study were collected from a topsoil (0–20 cm) at the Science and Education Park of Henan Agricultural University, located in Yuanyang County, Henan Province, China (N 35° 11′, E 113° 95′). The collected soil was alkaline (pH = 7.92) and contained alkali-hydrolyzale nitrogen 23.60 mg kg^-1^, available phosphorus 5.20 mg kg^-1^, available potassium 39.03 mg kg^-1^, organic matter 6.18 g kg^-1^, total Cd 0.19 mg kg^-1^ and total Se 0.18 mg kg^-1^. After air drying, the soil samples sieved using a 2 mm mesh and further autoclaved at 121 °C for 2 h. Secondly, CdCl_2_ 5H_2_O aqueous solution was added to the soil and kept at about 60% of the field water holding capacity (WHC) for pot experiment. Soil was stabilized for 14 days with 10 mg kg^−1^ Cd content. After Cd aging, each pot contained 800 g of soil, along with the root bag contained 400 g soil. The root bag was made of nylon mesh (mesh size 30 μm), allowing mycelium rather than wheat roots to pass through the root bag.

### Experimental design

The experiment included two Se treatments (0 and 5 mg kg^-1^) and three AMF inoculation treatments including non-mycorrhizal, *Rhizophagus intraradices* (Ri) and *Funneliformis mosseae* (Fm) (hereafter referred to as CK, Ri and Fm, respectively). These two kinds of AMF were purchased from the Institute of Root Biology, Yangtze University. There were six treatments with three replications, namely, CK, Se, Fm, Ri, Se + Fm and Se + Ri. Urea, potassium dihydrogen phosphate and potassium chloride were applied as N, P, K fertilizer sources, and the N, P_2_O_5_, K_2_O application rates were 0.20 g kg^-1^, 0.15 g kg^-1^ and 0.20 g kg^-1^, correspondingly, and Se fertilizer was Na_2_SeO_3_ 5H_2_O at an applied dosage of 5 mg kg^-1^. Before sown, Se fertilizer and base fertilizer were applied to soil in solution. The wheat seeds (*Triticum aestivum* cv. Bainong 207, purchased from Henan Agricultural High Tech Group Co., Ltd.) were sterilized for 15 min using a 10% H_2_O_2_ solution. Subsequently, they were rinsed three times with sterile water to ensure cleanliness and purity. AMF inoculation treatments were performed by adding 10 g AMF inoculum, and the non-AMF inoculation treatment was added with the same dose of autoclaved inoculation. Four wheat plants were planted in each pot, and watered by deionized water every two days to keep 60% WHC. After wheat emergence, all treatments were added to microbial washing solution of the original soil according to previous study [61].

Plant, rhizosphere and bulk soil samples were collected after planting for 10 weeks. Wheat samples were dried to a constant weight and then weighed. Rhizosphere soil samples were obtained by gently shaking the soil surrounding the roots to loosen it, leaving only approximately 1 mm of soil remaining. One portion of the rhizosphere and bulk soils was maintained at -80 °C, and the other portion was naturally air-dried.

### Determination of Cd and Se concentration

Wheat samples were digested with a mixture of HNO_3_ and HClO_3_ acids by graphite digester (LWY84B, Changzhou, China). Similarly, soil samples were digested with a mixed acid combination of HNO_3_ and HCl. Soil available Cd concentration was determined by diethylene triamine pentaacetic acid (DTPA) extraction method. The atomic absorption spectrophotometer (PinAAcie900T, Perkin Elmer, America) was employed for the determination of Cd content. In the case of Se determination, both wheat and soil samples were digested using a mixture of HNO_3_ and HClO_3_ acids. The atomic fluorescence spectrometer (AFS-933, Beijing, China) was then used for the subsequent analysis.

There are five chemical forms of Cd in soil can be divided: exchangeable form (referred to as EXC - Cd), carbonate bound (represented by CA − Cd), iron (Fe) -manganese (Mn) oxide bound (denoted as R_2_O_3_ − Cd), organic bound (represented by OM − Cd) and residual form (referred to as RES − Cd). To extract these forms, the first four forms were obtained using different solutions: 1 M MgCl_2_ (pH 7.0) for EXC-Cd, 1 M CH_3_COONa (pH 5.0) for CA − Cd, 0.04 M NH_3_OHCl (pH 2.0) for the R_2_O_3_ − Cd, and 0.02 M HNO_3_ + 30% H_2_O_2_ + 3.2 M CH_3_COONH_4_ for OM − Cd. The residues remained after extraction were transferred to a Teflon crucible and digested by adding HNO_3_ and HF to obtain RES-Cd [29]. An atomic absorption spectrophotometer (PinAAcie900T, Perkin Elmer, America) was used for the determination of Cd.

### Determination of soil microbial community

Bacterial and fungal communities were sequenced using the Illumina NovaSeq 6000 platform (Illumina, San Diego, CA, USA). The primers of 515 F and 806R were used for amplifying the V4 region of bacterial 16 S rRNA gene, and the primers of ITS1F and ITS2 were used to amplify the fungal ITS1 region [62]. The PCR reaction systems and conditions for bacteria and fungi were similar to those described in Walters et al. [63] and Gardes et al. [64], respectively. The PCR amplified products were purified using Vazyme VAHSTM DNA cleaning beads, and the Quant-iT PicoGreen dsDNA Detection Kit was utilized to quantify the results. The various quantitative processes were finished, the amplification products were combined in the appropriate ratios, and sequencing process utilized the NovaSeq 6000 SP kit on the Illumina NovaSeq platform. The raw sequencing data were stored in the Sequence Read Archive (SRA) of the NCBI database with PRJNA1013689 for bacteria and PRJNA1013134 for fungi.

The raw sequencing data were processed by QIIME2 2022.11 according to Bolyen et al. [65]. The data underwent a series of steps including frequency scrubbing, denoising, pairing, and removal of chimeric sequences by the DADA2 plugin according to Callahan et al. [66]. Subsequently, the obtained sequences were integrated based on 100% sequence similarity to generate distinct characteristic sequence (ASVs) and data sheets. Taxonomic identification of each ASV was conducted using the Sliva_132 and Unite_8 databases for bacteria and fungi, respectively. Abundance values below 0.001% were excluded from the dataset to facilitate streamlined analyses.

### Statistical analysis

SPSS 25.0 (IBM, Armonk, NY, America) was used for the statistical analysis. One-way analysis of variance (ANOVA) was conducted to examine the differences in the concentrations of Cd and Se in plants and soil, as well as the alpha diversity of microbes with P values lower than 0.05 were considered to be significant. All data were expressed as mean of three replications. To evaluate the dissimilarities in microbial communities, we utilized principal coordinate analysis (PCoA) and analysis of similarity (ANOSIM) calculated by the Bray-Curtis similarity matrix by the vegan package in R [67]. Linear discriminant analysis effect size (LEfSe) was used to analyze significant taxa at the phylum, family, and genus levels of soil fungal communities. The linear discriminant analysis (LDA) score was set at 2.0, and the *P* value was set at 0.05. Bar graphs were used to display significant LDA scores. The co-occurrence networks of bacteria and fungi were conducted in “humic” and “ggpolt2” software packages by R. Co-occurrence network models and images were produced using Gephi (version 0.9.2) software [68].

### Supplementary Information

**Supplementary Material 1.**

## Data Availability

The sequenced raw reads generated during the current study have been submitted to the National Center for Biotechnology Information (NCBI) with BioProject ID: PRJNA1013689 for bacteria and PRJNA1013134 for fungi. All data generated or analyzed during this study are included in this published article and its supplementary information files.

## Abbreviations

Ri: Rhizophagus intraradices
Fm: Funneliformis mosseae
