# Supplementary material for: Combined application of arbuscular mycorrhizal fungi and selenium fertilizer increased wheat biomass under cadmium stress and shapes rhizosphere soil microbial communities
Source: BMC Plant Biol. 2024 May 3;24:359. doi: 10.1186/s12870-024-05032-5 (PMC11067182; doi:10.1186/s12870-024-05032-5)
Supplement: Supplementary file 1 — Supplementary Material 1. [file 12870_2024_5032_MOESM1_ESM.docx]

**Supplementary Material**

**Combined application of arbuscular mycorrhizal fungi and selenium fertilizer increased wheat biomass under cadmium stress and shapes rhizosphere soil microbial communities**

Haiyang Liu^a,b 1^, Haoquan Wang^a,b 1^, Zhaojun Nie^a,b^, Zhikang Tao^a,b^, Hongyu Peng^a,b^, Huazhong Shi^c^, Peng Zhao^a,b^, Hongen Liu^a,b,^*

^a^College of Resources and Environment, Henan Agricultural University, Zhengzhou 450046, China

^b^Key Laboratory of Soil Pollution Control and Remediation in Henan Province, Zhengzhou 450046, China

^c^Department of Chemistry and Biochemistry, Texas Tech University, Lubbock, TX 79409, USA

^1^Authors with equal contribution.

*Corresponding author

The file includes:

Supplementary Table S1

Supplementary Figure S1 to S4

**Table S1.** The pH values in rhizosphere and bulk soils after wheat planting.

| Treatment | pH in  rhizosphere soil | pH in  bulk soil |
| --- | --- | --- |
| CK | 8.63±0.11d | 8.67±0.02b |
| Se | 8.65±0.02cd | 8.66±0.06b |
| Fm | 8.74±0.03bc | 8.71±0.01ab |
| Ri | 8.76±0.03ab | 8.72±0.04ab |
| Se + Fm | 8.82±0.06ab | 8.75±0.01a |
| Se + Ri | 8.84±0.08a | 8.73±0.09ab |


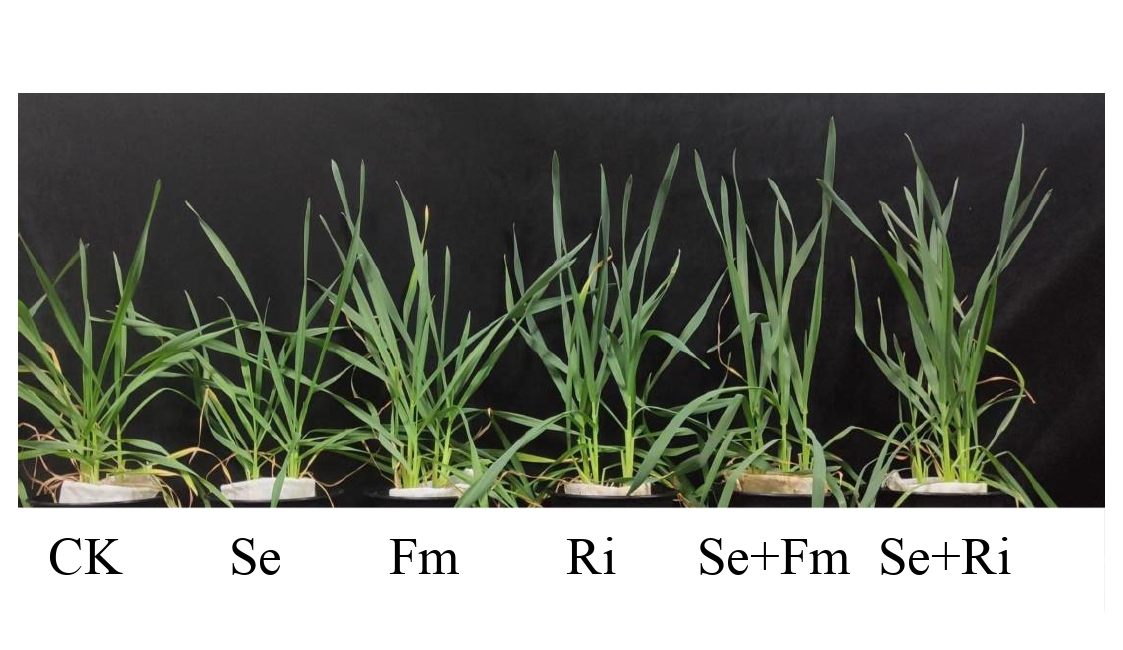


**Fig. S1.** The growth of wheat under different treatments after planting for 70 days.

**
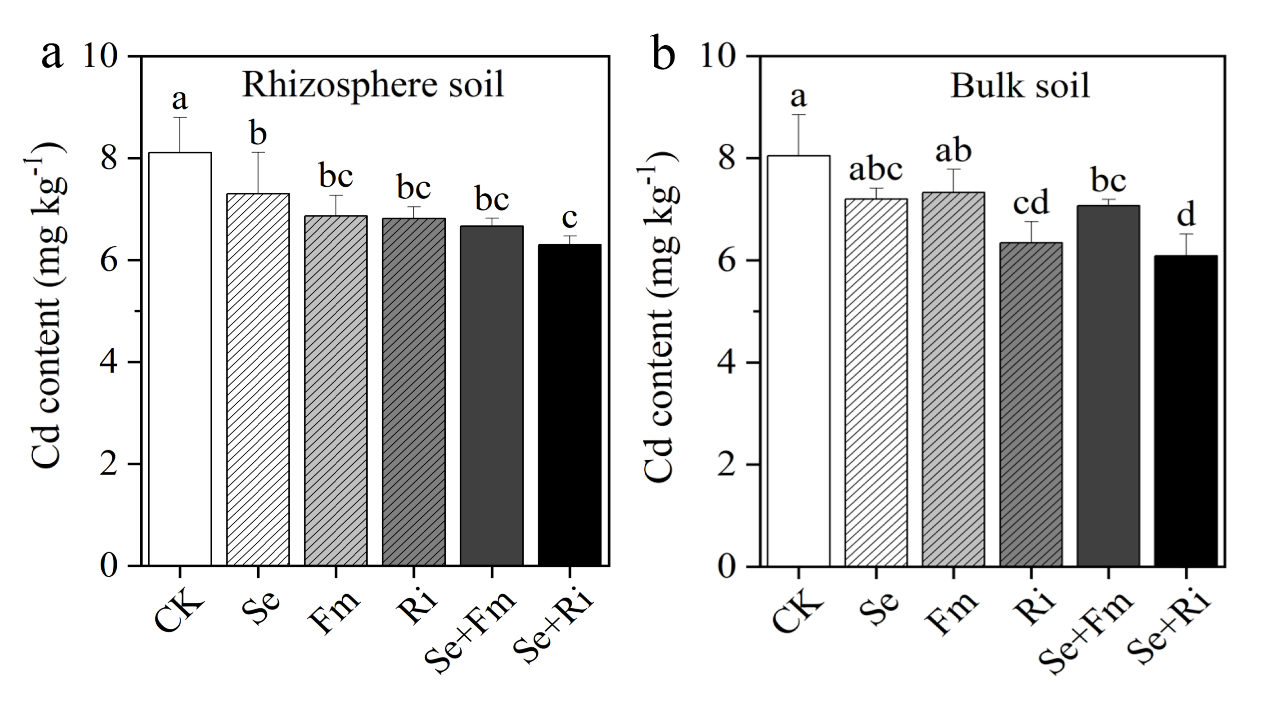
**

**Fig. S2.** Effects of different treatments on Cd concentrations in rhizosphere soil (a) and bulk soil (b) of wheat.


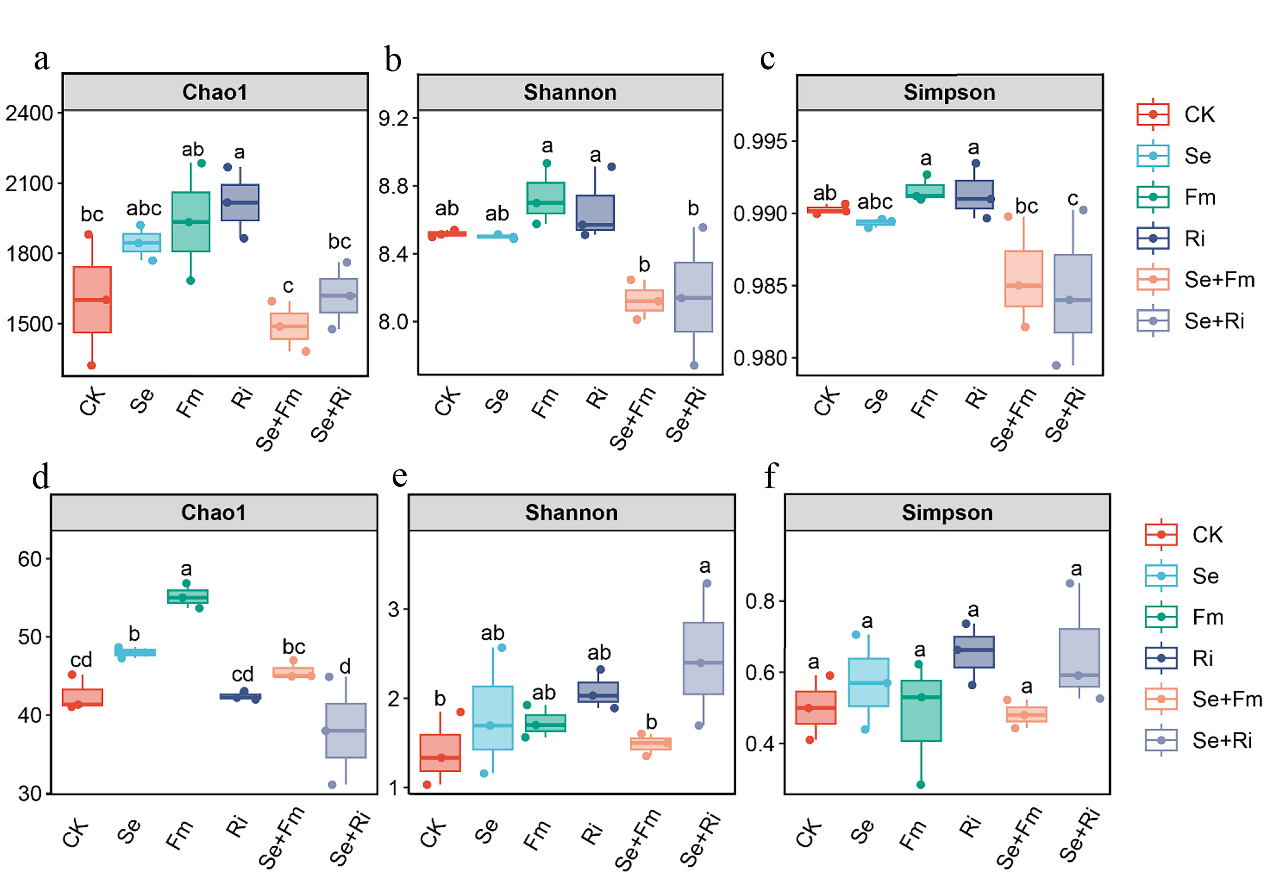


**Fig. S3.** Alpha diversity measurements of Chao 1 ( a and d ), Shannon index ( b and e ) and Simpson index ( c and f ) of bacteria ( a, b and c ) and fungi ( d, e and f ) under different treatments. Based on analysis of variance, different letters mean significant difference (*P* < 0.05).


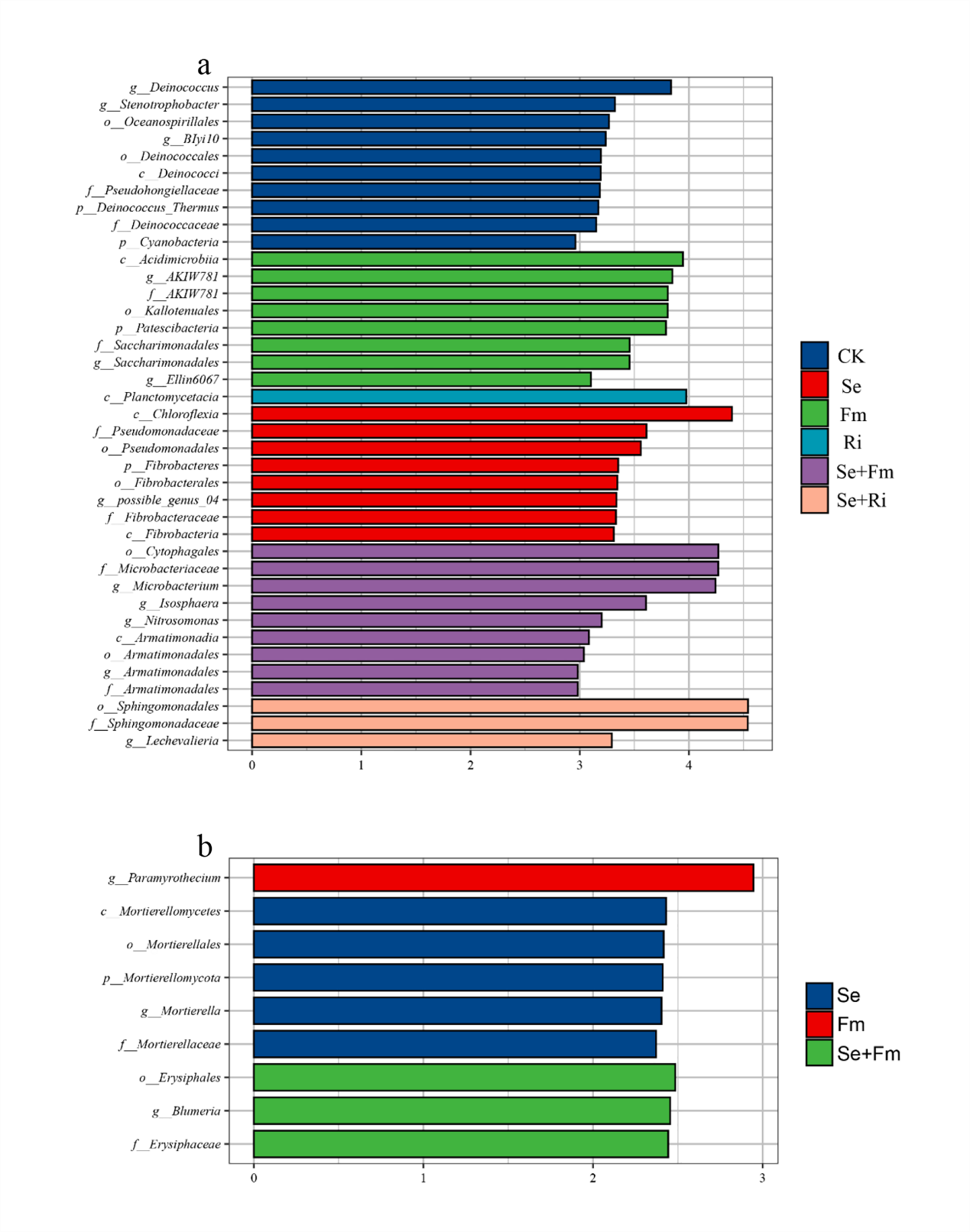
**Fig. S4.** Key phylotypes of bacterial (a) and fungal (b) communities responding to different treatments using LEfSe. The histogram shows the LDA scores computed for features (on the ASV level) with differential abundance under the Six treatments. Only taxa meeting a linear discriminant analysis with significance threshold of 2.0 for microbial communities are shown.
